# On-site blood culture incubation shortens the time to knowledge of positivity and microbiological results in septic patients

**DOI:** 10.1371/journal.pone.0225999

**Published:** 2019-12-11

**Authors:** Julika Schwarzenbacher, Sven-Olaf Kuhn, Marcus Vollmer, Christian Scheer, Christian Fuchs, Sebastian Rehberg, Veronika Balau, Klaus Hahnenkamp, Jürgen A. Bohnert, Matthias Gründling

**Affiliations:** 1 Department of Anesthesiology, University Hospital of Greifswald, Greifswald, Germany; 2 Institute of Bioinformatics, University Medicine Greifswald, Greifswald, Germany; 3 Department of Anesthesiology, Intensive Care, Emergency Medicine, Transfusion Medicine and Pain Therapy, Protestant Hospital of the Bethel Foundation, Bielefeld, Germany; 4 IMD Labor Greifswald, MVZ Labor Greifswald GmbH, Greifswald, Germany; 5 Friedrich Loeffler Institute of Microbiology, University Hospital Greifswald, Greifswald, Germany; Universita degli Studi di Parma, ITALY

## Abstract

**Introduction:**

To determine whether on-site incubation of blood cultures at the intensive care unit (ICU) improves not only the time to incubation but also time to positivity, time to knowledge of positivity and time to results (identification and antibiotic susceptibility testing).

**Methods:**

This observational single-centre study in ICU patients with severe sepsis and septic shock investigated the impact of blood culture incubation immediately on-site at the ICU (ICU group) by comparison with traditional processing in a remote laboratory (LAB group) on different time intervals of blood culture diagnostics from obtaining blood to clinician notification of final result. The effect of on-site incubation was evaluated in Kaplan-Meier estimates for the time to positivity, time to knowledge of positivity and time to microbiological results and a linear mixed model was built.

**Results:**

A total of 3,549 blood culture sets from 657 ICU patients were analysed: 2,381 in the LAB group and 1,168 in the ICU group. Overall, 660 (18.6%) blood culture sets were positive and 2,889 (81.4%) sets remained negative. On-site incubation was associated with reduced time to knowledge of positivity (46.9 h [CI 43.4–50.8 h] vs. 28.0 h [CI 23.6–32.2 h], p < 0.001) and reduced time to result (61.4 h [CI 58.4–64.8 h] vs. 42.1 h [CI 39.1–47.5 h], p < 0.001). In blood cultures processed instantaneously at the ICU compared to incubation in the remote laboratory within 4 h, the time to microbiological result was significantly reduced by 8.5 h (p < 0.001). Pre-existing anti-infective therapy had no significant impact on diagnostic time intervals.

**Conclusions:**

Instantaneous incubation of blood cultures in the ICU compared to incubation in a remote laboratory significantly improves time to knowledge to positivity and time to result. These effects are even more pronounced during off-hours of the microbiological laboratory. The results underline the importance of 24/7 diagnostics to provide round-the-clock processing of blood culture samples in patients with sepsis and septic shock and an immediate to communication of the results to the clinicians.

## Introduction

An adequate and early anti-infective therapy is crucial to improve the outcome of critically ill patients with sepsis and septic shock. Previous clinical data have shown an inappropriate or superfluous empirical antibiotic therapy in up to 40%, and up to 70% with fungemia before definitive identification of the pathogens detected [1–3].

Despite the low rate of positive pathogen detection in only up to 30% of the cases [4–8], blood cultures still represent the gold standard to diagnose bloodstream infections [9]. The current Surviving Sepsis Campaign guidelines recommend “that blood cultures be obtained prior to initiating antimicrobial therapy if cultures can be obtained in a timely manner” and subsequent incubation within 4 h after diagnosis [10]. Every hour of delayed incubation results in a decreased probability of culture positivity [11]. Recent studies have demonstrated an association of delayed antimicrobial administration with increased progression of sepsis to septic shock and increased mortality [12,13]. Unfortunately, the processing is time-consuming and typically takes 48–72 h for final results. However, even the knowledge of positivity of blood cultures without any specification of pathogens can already trigger the initiation of calculated anti-infective treatment. Results published by Prado et al. state that time to positivity of blood cultures are eligible for antibiotic de-escalation at 48 h [14]. Following definite pathogen identification and availability of its antimicrobial susceptibility, therapy can be specified, de-escalated or discontinued, respectively. De-escalation has been reported to be associated with improved outcome [15].

Because sepsis is a medical emergency, the reduction of time from taking blood cultures to final results is also a critical task of the microbiology laboratory to provide blood culture results as early as possible. However, only 20% of the microbiological laboratories in Europe offer a round-the-clock service. In Germany, on-site incubation takes 2 h, whereas the incubation in remote laboratories was delayed by at least 8 hours in more than 60% of all blood cultures [16].

Our objective is the comparison of immediate on-site blood culture incubation at the intensive care unit (ICU) with off-site blood culture incubation at a remote microbiological laboratory in patients with confirmed sepsis and septic shock to test the hypothesis that incubation immediately after obtaining blood cultures improves the time to incubation, time to positivity, time to clinician notification of positive results, time to knowledge of positivity and time to microbiological results, including pathogen identification and susceptibility testing.

## Materials and methods

### Ethical approval

The proposed clinical study was reviewed by the Ethics Committee of the University Medicine Greifswald (Identifier: BB 026/16). The Ethics Committee concluded that there are no ethical and legal reservations to perform the study according to the German Law. Therefore, the Ethics Committee grants approval for the proposed clinical study.

### Study design

In this observational single-centre study, ICU patients with severe sepsis and septic shock were analysed to study the impact of on-site incubation of blood cultures instantaneously at the ICU compared to off-site incubation at the following time intervals:

- Time to blood culture incubation (TTI): time from taking blood culture to start of incubation,
- Time to blood culture positivity (TTP): time from taking blood culture to positive growth signal,
- Time to knowledge of positivity (TTK): time from taking blood culture to time to notification of the attending physician about blood culture positivity, and
- Time to microbiological result (TTR): time from taking blood culture to the first available microbiological results, including pathogen identification and preliminary susceptibility results.

### Clinical setting

**Fig 1:**
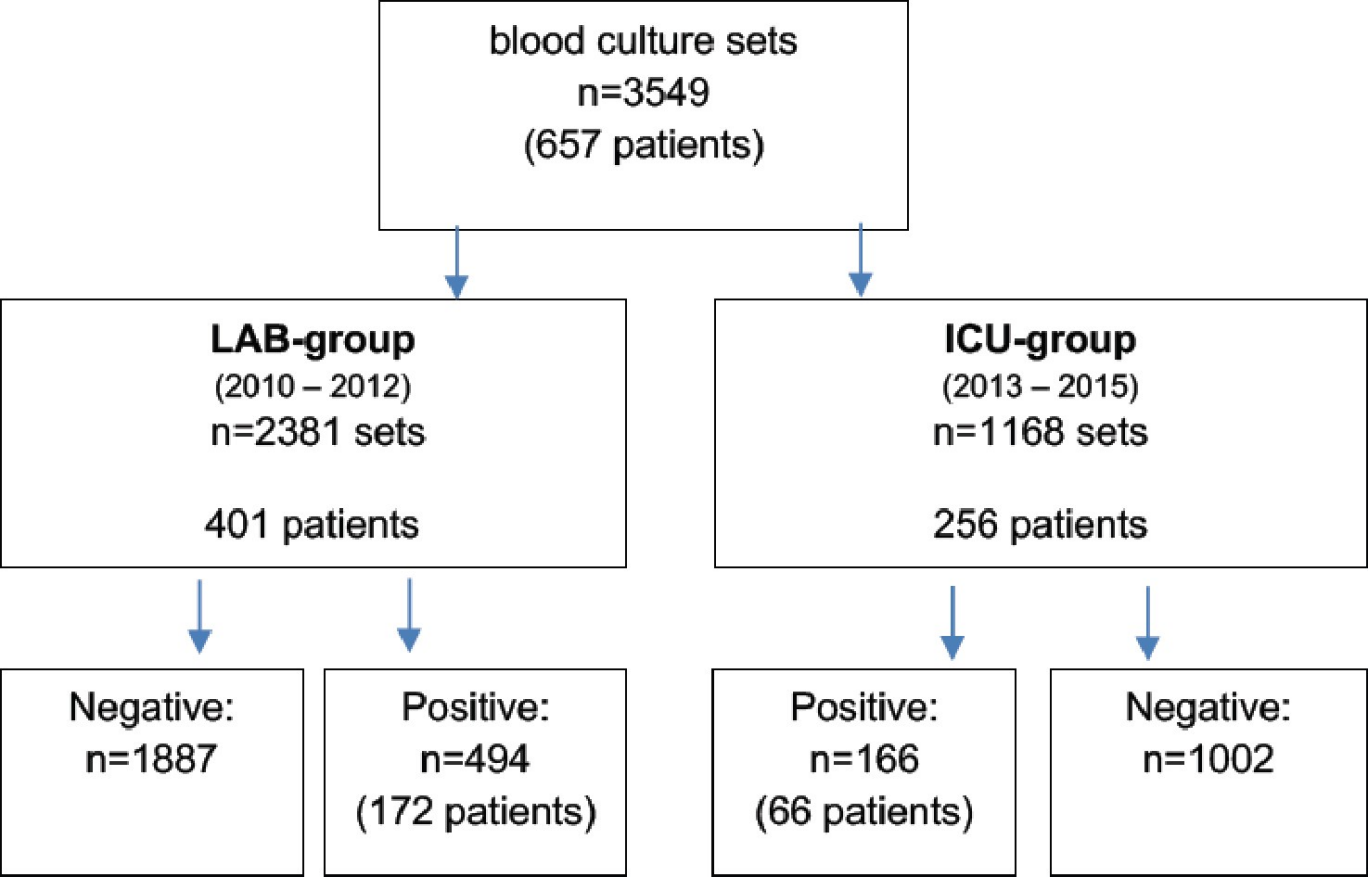
Flow chart of blood culture sets included. Blood cultures (n) were either incubated at the remote microbiology laboratory (LAB group) or incubated on-site at the intensive care unit (ICU group).

The study was conducted at the interdisciplinary 27-bed ICU at Greifswald University Hospital, Germany. We included blood cultures from patients with confirmed severe sepsis or septic shock [17] according to the American College of Chest Physicians/Society of Critical Care Medicine Consensus Conference(ACCP/SCCM) criteria [18] (Fig 1). Blood cultures were initially analysed in a remote microbiology laboratory connected to the ICU (LAB group) between January 2010 and December 2012. From September 2013 to December 2015, blood cultures were incubated immediately at the ICU within 1 hour (ICU group). Operating procedures for the incubator were validated, and the ICU staff was trained between the trial periods to load cultures 24/7. All blood culture sets are comprised of one aerobic and one anaerobic bottle (BACTEC™Plus Aerobic/F and BACTEC™Plus Anaerobic/F Culture Vials), which were incubated in BD BACTEC FX incubators (Becton-Dickinson USA) until cultures detected positive or for at least six days.

The microbiological laboratory where the diagnostic was performed was located 4 km away from the ICU in another building of the hospital. All blood culture bottles in the LAB group were submitted by car. The opening hours of the laboratory for routine processing were weekdays 7 am to 6 pm, Saturdays 7 am to 1 pm and Sundays and holidays 7 am to 12 noon, respectively. Routine blood culture deliveries were made during laboratory opening hours. In the case of transportation-related delays, blood cultures were stored at room temperature.

In the ICU group, blood cultures were incubated immediately after obtaining, directly at the ICU throughout the day. Positivity was alerted by an acoustic alarm. Positive blood culture bottles transported to the laboratory for Gram-stain and further analysis remained limited to the operating hours of the remote laboratory. In the case of positivity during the off-hours of the laboratory, cultures remained incubated until the next transport was available.

Positive cultures were Gram-stained, streaked onto Columbia sheep blood agar, chocolate agar, MacConkey lactose agar and Schaedler agar (Becton-Dickinson USA) for overnight incubation at 37°C. Species identification was then carried out using matrix-assisted laser desorption ionization time-of-flight mass spectrometry (MALDI-TOF) on a VITEK®MS device (bioMérieux, France) and susceptibility testing was performed using the VITEK®2 device (bioMérieux, France). The microbiologist on duty confirmed microbial growth according to Gram-stain by telephone call. If Gram-stain did not confirm bacterial growth, blood cultures were further incubated in the laboratory and Gram-stains were repeated daily. Final results were transmitted electronically and rendered for each blood culture set, including the results of the aerobic and anaerobic bottle. Therefore, our analysis focuses on blood culture sets and not on individual bottles.

### Statistical analysis

Statistical analysis was performed using R (R Foundation for Statistical Computing, R version 3.4.4; http://www.R-project.org). Baseline characteristics of patients and blood cultures were summarized in terms of means with standard deviation and in terms of quantity and proportion. Group comparisons were conducted by the two-tailed Welch’s t-test, Fisher’s exact test and Chi-Squared test accordingly.

Variations in processing durations were summarized and presented by the restricted means with 95% confidence intervals (CI). Group comparison was performed by time-to-event analysis. Consequently, we split the LAB group into three subgroups depending on the TTI (incubation groups: TTI ≤ 4 h, 4 h < TTI ≤ 8 h, TTI > 8 h) to study the effect of delayed incubation in the same cohort. We identified censored data in the TTK when no telephone call was made due to strains in the patient which already known. The TTK is, therefore, considered right-censored and the duration was considered to be at least TTP plus the time to the next business hour of the laboratory. The Kaplan-Meier estimator was used to estimate the time to event functions and logrank tests were used for comparison.

Because of non-proportional hazards and the quasi-normally distributed TTR, we decided to build a linear mixed model rather than a Cox regression model to explain the TTR by independent features, such as the incubation group, TTP, existing anti-infectives at sampling, and whether the microbiology was open at the time of growth detection.

## Results

A total of 3,549 blood culture sets from 657 sepsis patients (401 in the LAB group, 256 in the ICU group) were processed: 2,381 sets in the LAB group and 1,168 sets in the ICU group. Baseline patient characteristics for both groups in patients with positive blood cultures are tabulated in Table 1.

**Table 1:** Baseline characteristics of sepsis patients with positive blood cultures processed either in the laboratory (LAB group) or at the ICU (ICU group).

**Patients**	**LAB group n = 172**	**ICU group n = 66**	**P value**
Age in years, mean ± SD	65.7 ±12.5	68.7 ±11.4	0.080
Severe sepsis^1^, # (%)	30 (17.4%)	17 (25.8%)	0.151
Septic shock^2^, # (%)	142 (82.6%)	49 (74.2%)	
Abdominal infection, # (%)	67 (39.0%)	29 (43.9%)	0.828
Respiratory site infection, # (%)	34 (19.8%)	10 (15.2%)	
Other focus, # (%)	62 (36.0%)	24 (36.4%)	
Unknown focus, # (%)	9 (5.2%)	3 (4.5%)	
**Blood cultures**	**LAB group n = 2381**	**ICU group n = 1168**	**P value**
Blood culture sets with growth detection, # (%)	494 (20.7%)	166 (14.2%)	<0.001
Anti-infective treatment prior to sampling in blood culture sets with growth detection, # (%)	351 (71.1%)	115 (69.3%)	0.694

SD = standard deviation.

^1^ patients diagnosed with severe sepsis (suspected or present infection plus at least two of the following: heart rate > 90 beats per minute, respiratory rate >20 per minute, white blood cell count <4000/mm^3^ or >12000/mm^3^ or > 10% banded neutrophils, temperature < 36 or > 38°C).

^2^ patients diagnosed with septic shock (fulfilling criteria of severe sepsis plus signs of organ dysfunction).

Overall, 660 (18.6%) blood cultures had positive results: 494 (172 patients) in the LAB group and 166 (66 patients) in the ICU group. The positivity rate was higher in the LAB group (20.7% vs. 14.2%, p < 0.001) (Table 1). Contamination was found in 38 cases (27 in the LAB group, 11 in the ICU group). We found no significant differences in the contamination rates and pathogen distribution. However, we found more multimicrobial blood cultures in the LAB group (15.5% vs. 4.6%, p = 0.02) (Table 2).

**Table 2:** Distribution of pathogen isolates of blood cultures processed either in the laboratory or at the ICU.

Sets collected within 24 hours were combined	LAB group n = 227	ICU group n = 76	P-value
Contaminated sets°, # (%)	27 (11.9%)	11 (14.5%)	0.552
**In non-contaminated sets**	**n = 200**	**n = 65**	
Gram positive, # (%)	125 (62.5%)	38 (58.5%)	0.561
Gram negative, # (%)	56 (28.0%)	14 (21.5%)	0.335
Anaerobic pathogens, # (%)	5 (2.5%)	4 (6.2%)	0.229
Fungi, # (%)	25 (12.5%)	6 (9.2%)	0.657
**In non-contaminated sets**			
Multimicrobial, # (%)	31 (15.5%)	3 (4.6%)	0.020
Multiresistant pathogens, # (%)	12 (6.0%)	7 (10.8%)	0.265
Reactive, # (%)	8 (4.0%)	5 (7.7%)	0.317

° contamination: Single detection of coagulase-negative *staphylococci* or *propionibacterium acne* in all sets collected within 24 h.

**Fig 2:**
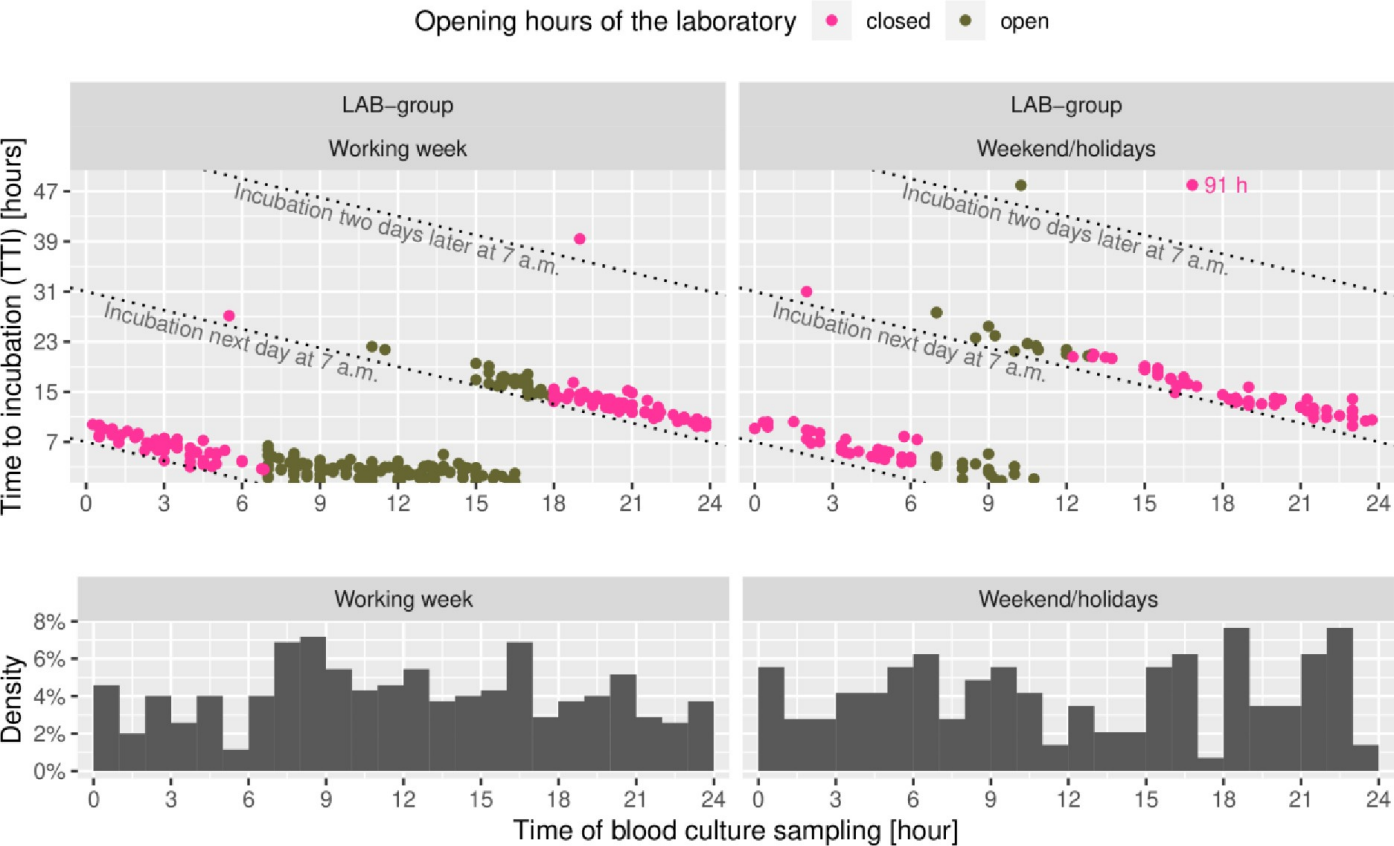
Time to incubation related to time of blood culture sampling (LAB group). Top: Magenta dots represent sampling during laboratory off-hours, green dots during working hours. Bottom: Overall distribution of sampling during working week and on weekends/holidays.

The overall distribution of samplings during weekdays compared to weekends/holidays and the TTI in relation to the time of sampling of the LAB group, showing an expected circadian distribution is illustrated in Fig 2.

**Fig 3:**
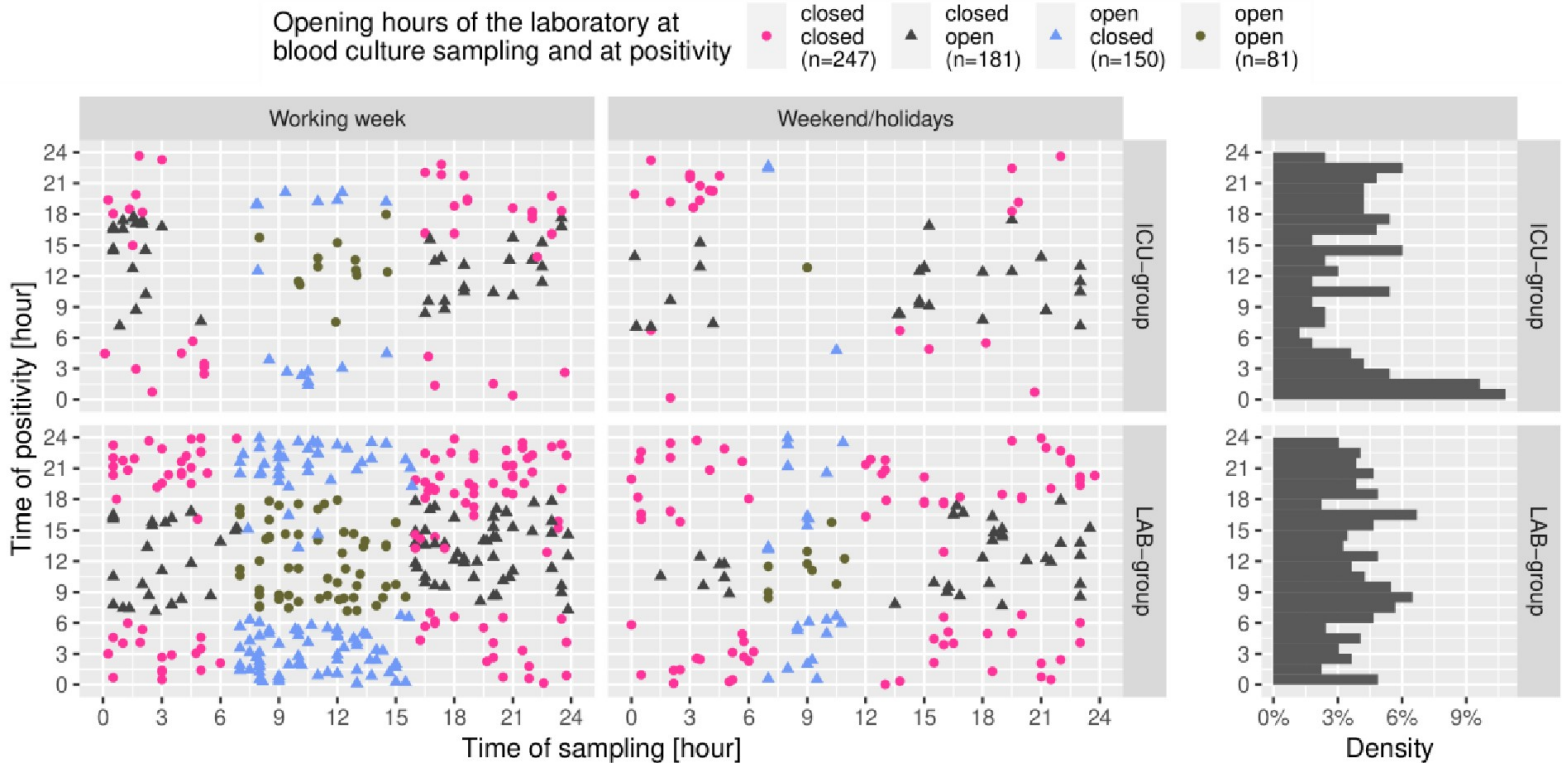
Overall distribution of sampling time vs. positivity time of blood cultures (LAB group and ICU group). Green dots represent sampling and positivity during laboratory opening hours. Blue triangles represent sampling during laboratory opening hours and positivity off-hours. Dark-grey triangles represent sampling during laboratory off-hours and positivity during opening hours. Magenta dots represent sampling and positivity during laboratory off-hours.

Fig 3 shows the overall distribution of sampling time in relation to the time of growth detection in both groups. The magenta dots (highest number of blood cultures) represent samplings with the largest delay between growth detection and further processing because these blood cultures flagged positively during the off-hours of the laboratory. Blood cultures obtained and flagged positive during laboratory opening hours show the most apparent time advantage (marked as green dots).

In the LAB group, 173 blood cultures (35. 0%) were incubated within 4 h, and 256 (51.8%) were incubated within 8 h, while all blood cultures in the ICU group were incubated within 1 h (Table 3). Because of the blood transportation, only 13 (2.6%) blood cultures in the LAB group were incubated within 1 h.

**Table 3:** Processing time of blood cultures processed in the laboratory and at the ICU.

	LAB group (n = 494)			ICU group (n = 166)	P-value***
	TTI ≤ 4 h (n = 173)	4 h < TTI ≤ 8 h(n = 83)	TTI > 8 h (n = 237**)		
Time to positivity (TTP), mean* (CI)	20.8 (19.1–22.9)	22.4 (20.9–24.7)	30.6 (27.9–34.0)	28.0 (23.6–32.2)	<0.001
Time to knowledge of positivity (TTK), mean* (CI)	44.7 (29.3–49.8)	36.0 (32.6–54.0)	47.8 (43.9–65.0)	28.0 (23.6–32.2)	<0.001
Time to first antimicrobial susceptibility results (TTR), mean* (CI)	50.6 (49.3–51.7)	57.5 (54.8–76.2)	65.3 (62.8–67.0)	42.1 (39.1–47.5)	0.005

* restricted mean duration in hours with 95% confidence interval.

** 1 observation deleted due to missing data.

*** P-value of logrank test.

**Fig 4:**
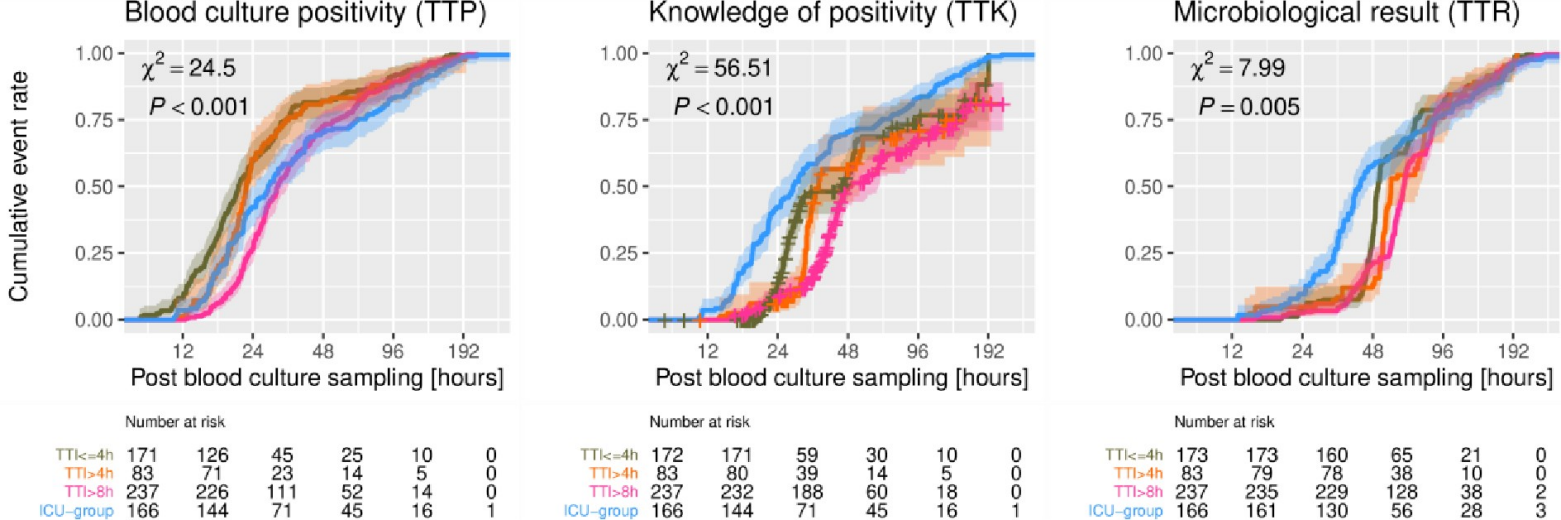
Kaplan-Meier estimates for TTP, TTK, and TTR. Cumulative event rates on logarithmic scale with 95% confidence bands (LAB group split according to TTI) with logrank test results.

Moreover, TTK and TTR differed significantly between the groups, as shown in Table 3. On-site incubation (ICU group) was associated with a reduced TTK (46.9 h [CI 43.4–50.8 h] vs. 28.0 h [CI 23.6–32.2 h], p < 0.001) and a reduced TTR (61.4 h [CI 58.4–64.8 h] vs. 42.1 h [CI 39.1–47.5 h], p < 0.001). These effects were even more pronounced within the first 48 h (Fig 4). Finally, TTR was also still significantly shorter (8.5 h, p<0.001) when sampling was processed directly at the ICU compared to instantaneous incubation in the laboratory (TTI ≤ 4 h), as suggested by the linear mixed model (Table 4). Additionally, logrank testing revealed that the TTP was significantly different in the ICU group compared to the entire LAB group (p = 0.03). Furthermore, splitting the LAB group into subgroups, we observed significantly prolonged TTP in bottles that were incubated beyond 8 hours (p < 0.001, cf. Table 3). It is noteworthy that there was no significant difference in TTR regarding pre-existing anti-infective therapy in the multivariable-adjusted regression model.

**Table 4:** Results of the linear mixed model for TTR. Each row lists the estimated, multivariable adjusted impact on the TTR. Estimates in hours are given for each predictor with standard error and p-value.

	Estimate (hours)	Standard error	P-value
(Intercept)	38.82	2.45	< 0.001
LAB group (TTI ≤ 4h)	(reference)		
LAB group (4h < TTI ≤ 8 h)	1.93	3.57	0.589
LAB group (TTI > 8 h)	-0.82	2.69	0.762
ICU group	-15.18	2.96	< 0.001
Microbiology open at growth detection	-11.44	2.16	< 0.001
No anti-infective therapy at sampling	-0.55	2.32	0.813
Time to positivity (TTP)	1.02	0.03	< 0.001

## Discussion

Immediate blood culture incubation significantly reduces the TTK and TTR. We found the TTK decreased by 18.9 h and the TTR decreased by 19.3 hours if the bottles were incubated directly at the ICU compared to a remote laboratory. These findings correspond with results published by Kerremans et al. [19]. Weinbren et al. reported a reduction in the time taken to detect most blood culture isolates to < 12 h by siting the incubator in the blood sciences laboratory and optimizing the pre-analytical and analytical phases of blood culture management [20]. In our study, the delay during the pre-analytic period, caused mainly by submission to the microbiological laboratory and limited opening hours, was associated with prolonged TTK and TTR.

The TTI and TTP we found in our study are within the range of previous studies [21–23]. Ronnberg et al. revealed a median TTI of 9 h in a mixed patient population compared to the 6.8 h we found [23]. Similarly, the increased TTI on weekends has been described before. Other studies found shorter transportation periods of 3 to 4 h; however, these did not report corresponding turnaround times [9–11]. Kerremans et al. reported a reduction of TTP defined as the time from collection to growth detection by 10 h if blood cultures were incubated immediately and continuously monitored [19]. Surprisingly, in our study, we found no time advantage for the TTP between the groups but a significantly increased TTP in the LAB group if TTI exceeds 8 hours. Though one should consider that TTP may be challenging to interpret, provides indirect information on the biomass and is associated with many other confounding factors (e.g. blood volume per bottle, conditions of cultures, transportation time, concomitant anti-infectives or sample type) [24,25]. Previous studies have demonstrated that a delay in loading the bottles into the incubator decreases the TTP, which was calculated by subtracting the time of receipt in the laboratory from the time required to detect a positive culture [26]. That may explain why we observed no effect on the TTP in the ICU group. However, because of the historical design of our study, we cannot exclude an analytical bias.

Current recommendations suggest blood culture incubation within 2 to 4 h [11,27]. Whereas only about one-third of all positive blood culture sets in the LAB group were incubated within 4 h, all sets in the ICU group were processed according to these recommendations within 1 h. In accordance with recently published data, we found an overall positivity rate of 18.6% [11,28]. Interestingly, there was a 6.5% higher positivity rate in the LAB group. One possible explanation is the higher proportion of shock patients in this group. Another explanation might be the higher proportion of multimicrobial blood cultures. Furthermore, this could be an effect confounded by our quality initiative, leading to an earlier admission of patients at risk for or at a previous stage of sepsis. We observed no difference in anti-infective treatment prior to sampling in blood culture sets with growth detection. We consider it unlikely that it is due to better bundle compliance because the latter was initiated two years before and monitored continuously by study nurses.

The TTK is the critical determinant of clinical relevance. It defines the interval until the clinician can react. Gehring et al. reported an increased rate of adequate antibiotic treatment from immediate communication in a prospective study on the effect of time-shifted telephone reporting of blood culture microscopy, despite some limits [29]. An isolated reduction in TTI or incubation time may not influence the treatment regime. A shorter TTK is a decisive clinical advantage of on-site culturing. The increased difference in TTK compared to TTI may partly be explained by the fact that the alarm of the incubator is immediately recognized by the ICU staff with on-site culturing, while additional information of the microbiologist to the clinician is required with external culturing. The relevance of this finding is emphasized by reports suggesting that mortality in patients with septic shock increases every hour with delayed effective antibiotic treatment [12,30]. It needs to be considered that the initial empirical antibiotic therapy is ineffective in 20–30% of all patients [12,31].

Idelevich et al. found only 13% of laboratories in 25 European countries provide 24-h service to start immediate processing of blood culture bottles that have flagged positive [32]. Results of a recent qualitative survey demonstrate that the majority of LABs in France, Germany, Italy and the UK are closed overnight and only about 40% offer services on weekends except for the UK (where 62% opened during weekends) [16]. However, prolonged storage at room temperature of blood cultures may lead to falsified test results and deferred processing to a delay in adequate antimicrobial therapy for patients with bloodstream infections [33–35]. The study of Morton et al. demonstrates that blood culture yield is lower at the weekend caused by delays or errors in incubation and processing (so-called “weekend effect”) [36]. An Italian study found a lower probability of culture positivity for each hour of delayed incubation. Additionally, there is evidence that the positivity rate decreases if TTI exceeds 24 h [22,37].

Although we were not able to abandon the transport time completely, because the microbiology laboratory was not on site in our setting, there was a significant time-saving effect in TTR by blood culture incubation within 4 h. Immediate blood culture incubation and short transportation routes of positive cultures for microbiological analysis may have a significant effect on improving patients’ outcome. Additional studies are necessary to evaluate the clinical significance of shorter TTP and TTK. However, the conveyance of data seems to be more important than on-site incubation, as suggested by a wider variety of TTK than TTP. Results could also be transmitted electronically to the clinician.

An alternative to reduce re-evaluation times could be the implementation of an on-site blood culture incubation system or rather a round-a-clock service for immediate loading of the incubator with blood cultures. The advantage discussed becomes even more evident during the off-hours of the microbiological laboratory. However, installing an on-site blood culture incubation system operated by the medical staff of the ICU is linked to increased workload and the stringent necessity of profound knowledge. We found that all staff members involved accepted the additional workloads as soon as they were sufficiently trained to operate the system. The logistic effort of extensive training required seems to be reasonable considering the time-saving effect. Nevertheless, a blood culture incubation system available at a permanently open laboratory with continuous sample receipt, the direct transmission of the findings to the clinician by phone or alert system, and immediate further analyses remains the best solution which, in turn, entails the requirement of additional staff and subsequently increases costs. Rapid diagnostic technologies based on PCR followed by electrospray ionization mass spectrometry allow a culture-independent pathogen detection in bloodstream infections. Although the spectrum of detectable pathogens is currently limited, molecular tests are a valuable add-on to conventional blood culture. Several studies observed a higher diagnostic yield than cultures [38–41].

The present study has some limitations. Our results and conclusions may not be generalizable because of the retrospective, single-centre design. Our study results are probably not applicable to hospitals with microbiological laboratories that are staffed 24 h a day, but it is anything but the rule. Both groups have been sampled during different years. This represents a potential confounding variable we cannot control for. Furthermore, we cannot exclude an analytical bias concerning the processing in the laboratory.

In conclusion, our study demonstrates that on-site incubation of blood cultures significantly shortens the time to knowledge of positivity and time to result. These effects are even more pronounced outside the processing time of the microbiological laboratory. The results underline the importance of round-the-clock processing of blood cultures in patients with sepsis and septic shock and to communicate the results to the clinician immediately.

## Supporting information

S1 Fig **A and B Kaplan-Meier cumulative event rates.** The figure displays the Kaplan-Meier estimator with a 95% confidence band for turnaround times stratified for antibiotic therapy (A) and sepsis severity (B). The horizontal axis displays the time post blood culture sampling. The vertical axis displays the cumulative event rate.
(DOCX)
