# Supplementary material for: On-site blood culture incubation shortens the time to knowledge of positivity and microbiological results in septic patients
Source: PLoS One. 2019 Dec 11;14(12):e0225999. doi: 10.1371/journal.pone.0225999 (PMC6905563; doi:10.1371/journal.pone.0225999)
Supplement: S1 Fig — A and B Kaplan-Meier cumulative event rates. The figure displays the Kaplan-Meier estimator with a 95% confidence band for turnaround times stratified for antibiotic therapy (A) and sepsis severity (B). The horizontal axis displays the time post blood culture sampling. The vertical axis displays the cumulative event rate. (DOCX) [file pone.0225999.s001.docx]

### S1 Fig. Kaplan-Meier cumulative event rates.

Figure displays the time to positivity stratified for antibiotic therapy and severe sepsis and septic shock. Horizontal axis displays the time post blood culture sampling. Vertical axis displays the cumulative event rate of blood culture samples flagged positive.
